# GABA_*A*_/Benzodiazepine Receptor Complex in the Dorsal Hippocampus Mediates the Effects of Chrysin on Anxiety-Like Behaviour in Female Rats

**DOI:** 10.3389/fnbeh.2021.789557

**Published:** 2022-01-05

**Authors:** Juan Francisco Rodríguez-Landa, Fabiola Hernández-López, Lucía Martínez-Mota, Damiana Scuteri, Blandina Bernal-Morales, Eduardo Rivadeneyra-Domínguez

**Affiliations:** ^1^Laboratorio de Neurofarmacología, Instituto de Neuroetología, Universidad Veracruzana, Xalapa, Mexico; ^2^Facultad de Química Farmacéutica Biológica, Universidad Veracruzana, Xalapa, Mexico; ^3^Instituto Mexicano del Seguro Social, Unidad de Medicina Familiar No. 66, Xalapa, Mexico; ^4^Dirección de Investigaciones en Neurociencias, Instituto Nacional de Psiquiatría Ramón de la Fuente Muñiz, Mexico City, Mexico; ^5^Pharmacotechnology Documentation and Transfer Unit, Section of Preclinical and Translational Pharmacology, Department of Pharmacy, Health and Nutritional Sciences, University of Calabria, Rende, Italy; ^6^Regional Center for Serious Brain Injuries, S. Anna Institute, Crotone, Italy

**Keywords:** allopregnanolone, anxiety, flavonoid, GABA_*A*_ receptor, ovarian cycle

## Abstract

Systemic injections of the flavonoid chrysin (5,7-dihydroxyflavone) exert anxiolytic-like effects in ovariectomised and cycling female rats through actions on gamma-aminobutyric acid-A (GABA_*A*_) receptors; however, it is unknown if chrysin directly acts on brain structures that are involved in regulating emotional processes, such as the hippocampus. The present study evaluated the effects of intrahippocampal microinjections of 0.25, 0.5, and 1 μg of chrysin on anxiety-like behaviour in the elevated plus maze (EPM) and locomotor activity test (LAT) in female rats in proestrus and dioestrus. Similar doses of the neurosteroid allopregnanolone were used as a reference GABAergic anxiolytic drug. The participation of the GABA_*A*_/benzodiazepine receptor complex was evaluated by administering the antagonists picrotoxin, bicuculline and flumazenil. In proestrus, 0.5 and 1 μg of chrysin and allopregnanolone induced anxiogenic-like behaviour. In dioestrus, chrysin, and allopregnanolone (0.5 μg) induced anxiolytic-like effects. Picrotoxin, bicuculline and flumazenil prevented the effects of chrysin and allopregnanolone in both proestrus and dioestrus. None of the treatments significantly affected locomotor activity. These results indicate that the GABA_*A*_/benzodiazepine receptor complex in the dorsal hippocampus regulates the effects of chrysin on anxiety-like behaviour, similar to the actions of allopregnanolone. The divergent effects of treatments across the oestrous cycle phases suggest complex interactions between GABA_*A*_ receptors and compounds with an anxiolytic potential.

## Introduction

Some anxiety disorders affect a higher percentage of women than men worldwide (Piggott et al., 2019), which have been associated with hormonal, biochemical, psychological and sociocultural factors (Velasco et al., 2019). Fluctuations in plasma and brain concentrations of oestradiol, progesterone, and some of their metabolites during the ovarian cycle are related to females’ higher vulnerability to stressors that may trigger anxiety (Rodríguez-Landa et al., 2015; Stanikova et al., 2019). At the preclinical level, anxiety-like behaviour is higher during metoestrus-dioestrus, characterised by low concentrations of steroid hormones than proestrus-oestrus which, in contrast, presents high hormonal concentrations (Marcondes et al., 2001). This naturally occurring anxiety-like behaviour prompted the evaluation of substances that relieve anxiety associated with metestrus-dioestrus (Ravenelle et al., 2018; García-Ríos et al., 2019; Rodríguez-Landa et al., 2021), which is a physiological state similar to the premenstrual period in women (Lovick, 2008). In this way, some flavonoids exert anxiolytic-like effects in rodents. The systemic administration of chrysin (5,7-dihydroxyflavone) produces anxiolytic-like effects in male rats (Salgueiro et al., 1997; Zanoli et al., 2000), ovariectomised and cycling female rats (Rodríguez-Landa et al., 2019, 2021). Such effects are blocked by gamma-aminobutyric acid-A (GABA_*A*_) receptor complex antagonists, suggesting that chrysin shares similar mechanisms of action with anxiolytic drugs and neurosteroids (Crocetti and Guerrini, 2020). This flavonoid exerts neurochemical and neurotrophic effects in brain structures involved in the neurobiology of anxiety and depression, such as the prefrontal cortex and limbic cortex (Filho et al., 2015; Germán-Ponciano et al., 2021).

The GABA_*A*_/benzodiazepine receptor complex is widely distributed in brain structures involved in emotional processing (Pirker et al., 2000; Engin and Treit, 2007b; Albrecht et al., 2021) such as the hippocampus, which is a target of anxiolytic drugs (Farajdokht et al., 2020). Intrahippocampal microinjections of GABAergic compounds produce anxiolytic- and antidepressant-like effects that are blocked by GABA_*A*_ receptor antagonists (Nin et al., 2008; Rezvanfard et al., 2009; Rodríguez-Landa et al., 2009; Farajdokht et al., 2020). However, it is unknown if the hippocampus modulates anxiolytic-like effects produced by the flavonoid chrysin in cycling female rats via GABAergic actions. Previously, we have demonstrated that specific doses of picrotoxin, bicuculline, and flumazenil (all injected i.p.) antagonise the anxiolytic-like effects of GABAergic compounds without exerting intrinsic activity (Rodríguez-Landa et al., 2013, 2019); which permits their use as a pharmacological tool to explore the potential mechanism of action of GABAergic compounds with anxiolytic effects. Therefore, we extend our knowledge of the participation of the hippocampus, where the *Cornus Ammonis* 1 (CA1) area plays a critical role in anxiety-like behaviour through ligands that act on the GABA_*A*_ receptor (Rezayat et al., 2005; Jimenez et al., 2018).

We hypothesised that a microinjection of chrysin in the CA1 area of the dorsal hippocampus reduces anxiety-like behaviour in dioestrus through the GABA_*A*_/benzodiazepine receptor complex. GABA_*A*_ receptor antagonists such as picrotoxin, bicuculline, and flumazenil were used to explore the participation of chloride ion channels, the recognition site for GABA and the benzodiazepine binding site, respectively, in the effects of chrysin on anxiety-like behaviour. The effects of chrysin were compared with those of similar doses of the neurosteroid allopregnanolone, which was used as a reference anxiolytic GABAergic drug (Bitran et al., 1991).

## Materials and Methods

### Ethics

All experimental procedures were performed in strict accordance with the Guide for the Care and Use of Laboratory Animals (National Research Council, 2011) and *Norma Oficial Mexicana para el Cuidado y Uso de Animales de Laboratorio* (NOM-ZOO-062, 1999). All efforts were made to minimise animal discomfort and reduce the number of animals according to the Reduce, Refine, Replace (3R) principles of preclinical research (Russell et al., 2005). The general protocol was approved by the institutional internal committee for the care and use of laboratory animals.

### Animals

Adult female Wistar rats aged 12–13 weeks (250–280 g) were included in the study. The rats were bred in the vivarium of the Faculty of Biological Pharmaceutical Chemistry of Universidad Veracruzana (Xalapa, Veracruz, México) and housed separately from male rats beginning at weaning. The rats were housed in Plexiglas cages, 4–5 per cage (44 cm width × 33 cm length × 20 cm height) in a room under a 12-h/12-h light/dark cycle (lights ON at 7:00 AM) and an average temperature of 25 \°C (± 2°C). Animals had *ad libitum* access to purified water and food (Nutri-cubos Purina, Agribrands Purina, Ciudad de México, México) during the study period, except during the experimental intervention periods. All experimental sessions were conducted between 11:00 a.m. and 2:00 p.m.

The rats were randomly assigned to different groups using an online free program.^1^ The sample size per group (*n* = 8) was based on previous studies that found that 7–8 rats per group (Estrada-Camarena et al., 2019; Puga-Olguín et al., 2019; Rodríguez-Landa et al., 2019) were sufficient to detect anxiolytic-like effects in the behavioural tests used here without compromising the statistical power. This is also supported by the 3R principles of preclinical research (Russell et al., 2005).

### Drugs

Solutions of chrysin and allopregnanolone were prepared daily in 35% 2-hidroxypropyl-γ-cyclodextrin and administered intra-hippocampus in a volume of 0.3 μL per rat. Picrotoxin, bicuculline, and flumazenil were freshly prepared in 0.9% NaCl and i.p. administered at a volume of 1 ml/kg. All reagents were purchased from Sigma-Aldrich Co. (St. Louis, MO, United States).

### Experimental Groups and Treatments

#### Experiment 1. Effects of Chrysin on Anxiety-Like Behaviour

**FIGURE 1:**
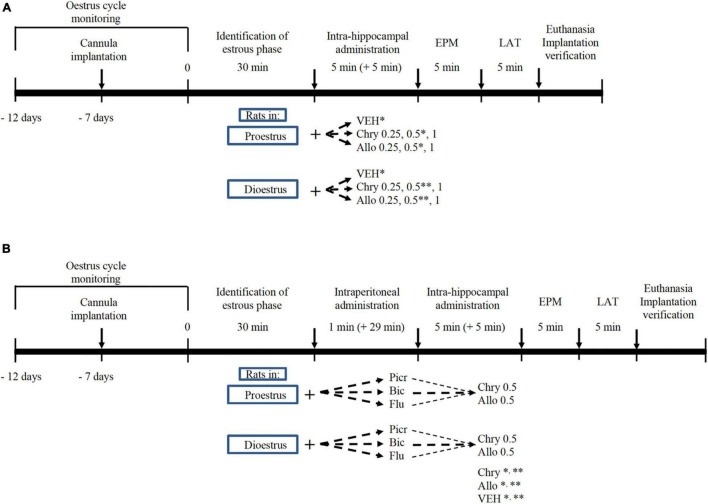
Timeline of experimental interventions in oestrous cycling rats. Effect of chrysin (Chry) and allopregnanolone (Allo) in proestrus or dioestrus females. Fourteen groups were included (*n* = 8 rats per group). Chrysin and allopregnanolone were administered at 0.25, 0.5, and 1 μg **(A)**. Effect of GABA_*A*_/benzodiazepine receptor complex antagonists on actions of Chry and Allo in proestrus or dioestrus females **(B)**. Twelve antagonised groups were included with data of VEH, Chry and Allo at *proestrus and ^**^dioestrus from **(A)**. VEH, vehicle; Picr, picrotoxin; Bic, bicuculline; Flu, flumazenil; EPM, elevated plus maze; LAT, locomotor activity test.

Female rats were assigned to 14 independent groups (*N* = 112 rats): seven groups in proestrus and seven groups in dioestrus. For each phase group, rats received a single intra-hippocampal microinjection as follows: vehicle (35% 2-hidroxypropyl-γ-cyclodextrin solution), chrysin (0.25, 0.5, and 1 μg, respectively) and allopregnanolone (0.25, 0.5, and 1 μg, respectively). All rats were evaluated using the elevated plus maze (EPM) and locomotor activity test (LAT) as previously reported (Rodríguez-Landa et al., 2009). The dosage was based on a previous study, in which the intrahippocampal administration of 0.2 μg of allopregnanolone produced anxiolytic-like effects in the open-field test (Martín-García and Pallarès, 2005). In the present study, a wider dose range was assessed to identify possible anxiolytic-like effects in two paradigms, as illustrated in Figure 1A.

#### Experiment 2. Influence of GABA_*A*_ Antagonists on Chrysin-Induced Anxiolytic Activity

To evaluate the role of GABA_*A*_/benzodiazepine receptor complex in the anxiolytic-like effect of chrysin and allopregnanolone, the rats were assigned to 12 independent groups (*N* = 96): six groups in proestrus and six groups in dioestrus. For each oestrous phase, the animals received single i.p. injections of picrotoxin (1 mg/kg), bicuculline (1 mg/kg) or flumazenil (5 mg/kg), and 30 min later, they were microinjected intra-hippocampus with chrysin or allopregnanolone at the effective dose (0.5 μg/rat) identified in experiment 1. To reduce the number of animals according to the 3R principles (Russell et al., 2005), the proestrus and dioestrus rat groups that were microinjected with the anxiolytic dose of chrysin or allopregnanolone (0.5 μg) were taken from Experiment 1 and used for statistical analysis. All rats were evaluated in the EPM and LAT as illustrated in Figure 1B. Antagonist schedules were based on a previous study in which such doses (all of them i.p. injected) antagonised anxiolytic-like effects of GABAergic compounds, without producing any effects in the LAT and EPM (Rodríguez-Landa et al., 2013).

### Ovarian Cycle Phase

Oestrous cyclicity was monitored daily (10:00 A.M.) through vaginal lavages and using light microscopy, as described in previous studies (Guillén-Ruiz et al., 2021; Rodríguez-Landa et al., 2021). Only females with three consecutive 4-day oestrous cycles (i.e., proestrus, oestrus, metestrus, and dioestrus) were included in the study and randomised to treatments. This criterion was used such that rats would be in vaginal proestrus or dioestrus on the day of behavioural evaluation.

Proestrus was chosen considering that this phase corresponds with higher levels of oestradiol and moderate levels of progesterone, while dioestrus was associated with low levels of oestradiol and progesterone (Walmer et al., 1992; Goldman et al., 2007). The proestrus and dioestrus endocrine milieu have been consistently reported to lead rats to experience lower and higher levels of anxiety throughout the cycle (Estrada-Camarena et al., 2019; Rodríguez-Landa et al., 2021), which is in agreement with the objectives of the present study.

### Stereotactic Surgery

The rats were pre-treated with atropine sulphate (0.05 mg/kg, i.p., Sigma-Aldrich, St. Louis, MO, United States) and then deeply anaesthetised with sodium pentobarbital (80 mg/kg, i.p., Cheminova, Ciudad de México, México). Additionally, local analgesia was induced in the surgical area (lidocaine 0.5% spray, PiSA-Farmaéutica, Guadalara, Jal, Mexico). They were fixed in a stereotaxic frame (Stoelting, Wood Dale, IL, United States), and a stainless-steel guide cannula (10-mm length, 22 gauge) was unilaterally implanted in the left dorsal hippocampus (anterior/posterior, −3.8 mm; medial/lateral, −2.0 mm; dorsal/ventral, −2.0 mm) according to the Paxinos and Watson (2014) rat brain atlas. To minimise postsurgical pain, 50 mg/kg metamizole (Dipirona50, Virbac Animal Health, Guadalajara, Mexico) was intramuscularly administered 4 days post-surgery. The treatments and behavioural tests began 7 days after surgery.

### Microinjection

Chrysin, allopregnanolone or vehicle was microinjected in the dorsal hippocampus through a stainless-steel injector cannula (11-mm length, 30 gauge) connected by a polyethylene tube to a Hamilton microsyringe (10 μL) that was attached to a programmable infusion pump (KD Scientific, Holliston, MA, United States) that allowed controlled microinjection of 0.3 μL at a constant rate of 0.06 μL/min according to previous studies (Rodríguez-Landa et al., 2009). The microinjection lasted for 5 min. The injector cannula was left in place for an additional 5 min to allow diffusion and to avoid reflux.

To verify the microinjection site, at the end of the experiment, the rats were deeply anaesthetised with pentobarbital (80 mg/kg, i.p.). The guide cannula was microinjected 0.3 μL of Evans blue to mark the injection site, and then the brain was removed and immersed in 40% formaldehyde for 72 h to be sliced. The microinjection site was examined using a light microscope, and the Paxinos and Watson rat brain atlas was used as a reference.

### Behavioural Tests

The behavioural tests began 5 min after the drug microinjections were completed. The effects of the treatments on anxiety-like behaviour were first evaluated in the EPM (evaluated variables: time spent on the open arms, number of open-arm entries and number of closed-arm entries; 5-min session), and then in the LAT (evaluated variables: number of crossings, time spent grooming and time spent rearing; 5-min session) to discard motor effects, as described in Supplementary Material 1. All sessions were videotaped. Two blinded independent observers measured the variables using *the ex Professional* software program until > 95% agreement was reached among them.

### Statistical Analysis

The data were analyzed using the two-way analysis of variance (ANOVA), with treatment and the oestrous cycle phase as factors once the assumptions of normality and homogeneity were verified. The level of significance was set at *p* < 0.05. Values of *p* ≤ 0.05, as determined by ANOVA, were followed by the Student-Newman-Keuls *post hoc* test. The results are expressed as mean ± standard error. All analyses were conducted using SigmaPlot (version 12.0; Systat Software, Chicago, IL, United States).

## Results

### Verification of the Microinjection Site

Six rats were excluded from the statistical analysis. Three rats removed the implants before the experiment concluded, and three rats were incorrectly implanted and presented areas of oedema and hemorrhage that surrounded the microinjection site. The brain analysis of the remaining rats confirmed that the microinjection effectively targeted the dorsal hippocampus at the intended stereotactic coordinates approximately in anterior/posterior, −3.48 to −4.20 mm from bregma; medial/lateral, −2.0 mm; dorsal/ventral, −2.0 to −2.5 mm as illustrated in Supplementary Figure 1. Thus, the final data analysis included seven to eight rats per group.

### Effects of Chrysin on Anxiety-Like Behaviour

#### Elevated Plus Maze

**FIGURE 2:**
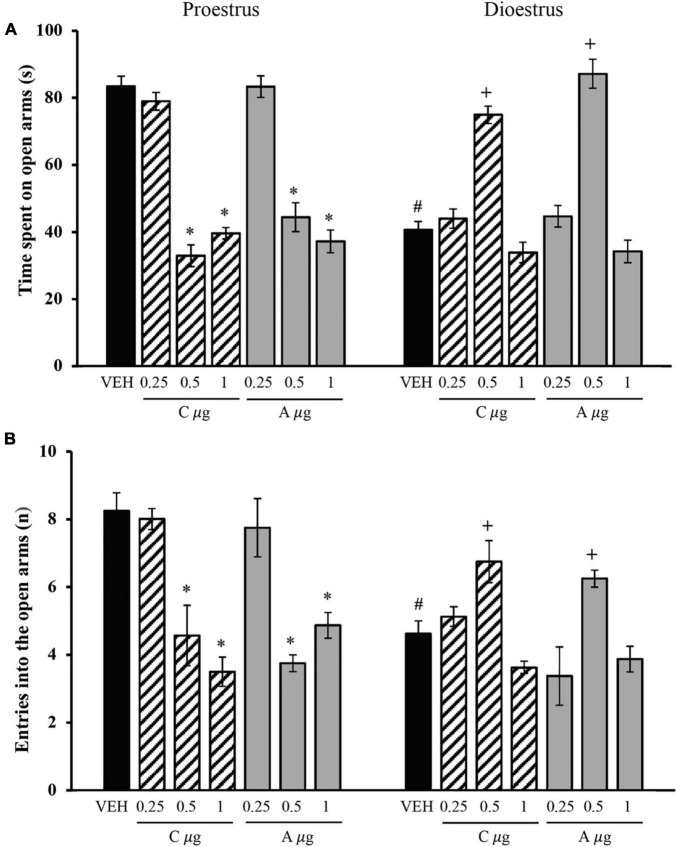
Effects of microinjections of chrysin and allopregnanolone in the dorsal hippocampus in the elevated plus maze in rats. **(A)** Time spent on open arms. **(B)** Number of entries into open arms. *p < 0.05, vs. vehicle and 0.25 μg chrysin and allopregnanolone in proestrus; ^#^*p* < 0.05, vs. VEH-proestrus; ^+^*p* < 0.05, vs. all groups in dioestrus (Student-Newman-Keuls *post hoc* test). VEH, vehicle; C μg, micrograms of chrysin; A μg, micrograms of allopregnanolone. The data are expressed as the mean ± standard error of the mean from 7 to 8 rats per group.

The analysis of the time spent on the open arms revealed significant effects of the treatment [*F*_(6_,_96)_ = 37.647, *p* < 0.001] and oestrous cycle phase [*F*_(1_,_96)_ = 13.017, *p* < 0.001] and a significant treatment × oestrous cycle phase interaction [*F*_(6_,_96)_ = 73.649, *p* < 0.001]. The *post hoc* test showed that the vehicle-dioestrus group exhibited significantly less time in the open arms compared with the vehicle-proestrus group. In proestrus, 0.5 and 1 μg of chrysin and allopregnanolone reduced this variable compared with the vehicle group and 0.25 μg of chrysin and allopregnanolone groups, reaching similar values as the vehicle-dioestrus group. No significant differences were found between 0.5 and 1 μg of chrysin and allopregnanolone during proestrus. In dioestrus, only 0.5 μg of chrysin and allopregnanolone significantly increased the time spent on the open arms compared with the vehicle-dioestrus group and 0.25 and 1 μg of chrysin and allopregnanolone groups (Figure 2A). The microinjection of 0.25 and 1 μg of chrysin and allopregnanolone did not significantly affect this variable.

The analysis of the number of entries into the open arms showed no significant effect of the treatment [*F*_(6_,_96)_ = 12.050, *p* = 0.183] but a significant effect of the oestrous cycle phase [*F*_(1_,_96)_ = 5.425, *p* = 0.022] and a significant treatment × oestrous cycle phase interaction [*F*_(6_,_96)_ = 14.267, *p* < 0.001]. The *post hoc* test showed that the vehicle-dioestrus group exhibited a significantly lower number of entries into the open arms than the vehicle-proestrus group. In proestrus, chrysin (0.5 μg) and allopregnanolone (1 μg) equally reduced the number of entries into the open arms compared with the vehicle group and 0.25 μg of chrysin and allopregnanolone groups, reaching similar values as the vehicle-dioestrus group. In dioestrus, only 0.5 μg of chrysin and allopregnanolone increased the number of entries into the open arms compared with the vehicle-dioestrus group and 0.25 and 1 μg of chrysin and allopregnanolone groups (Figure 2B). The microinjection of 0.25 and 1 μg of chrysin and allopregnanolone did not affect this variable in dioestrus.

The results of the closed-arm entries are presented in Supplementary Table 1. The statistical analysis did not reveal significant differences associated with treatments [*F*_(6_,_96)_ = 0.475, *p* = 0.826], the ovarian cycle phase [*F*_(1_,_96)_ = 0.350, *p* = 0.556], and the treatment × oestrous cycle phase interaction [*F*_(6_,_96)_ = 1.927, *p* = 0.084].

#### Locomotor Activity Test

The LAT results are presented in Supplementary Table 2. None of the variables in this test (i.e., crossings, grooming, and rearing) were significantly affected by the treatments or ovarian cycle phase.

## Influence of Gaba_*A*_ Antagonists on Chrysin-Induced Anxiolytic Activity

### Elevated Plus Maze

**FIGURE 3:**
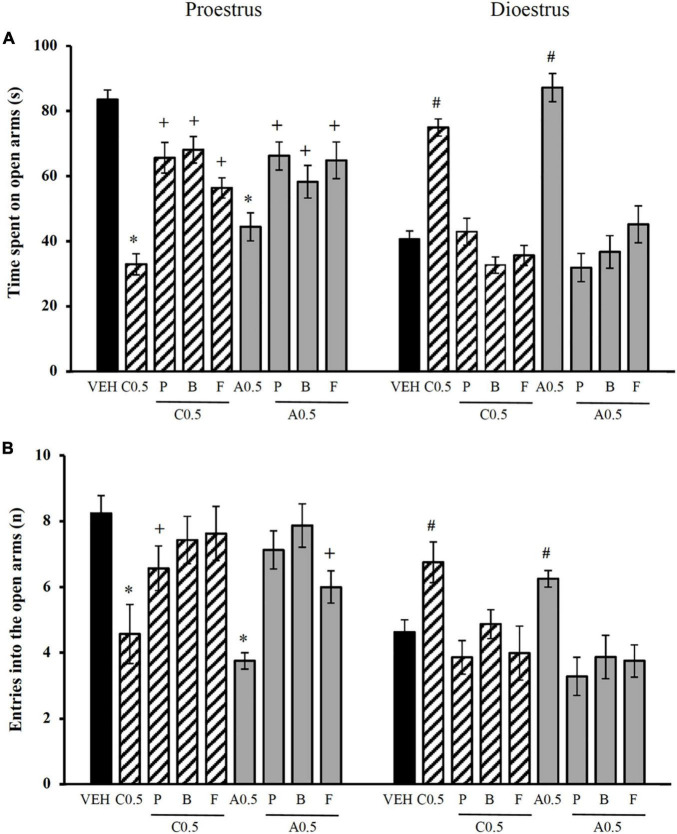
Influence of GABA_*A*_ receptor antagonists on the effects of microinjections of chrysin and allopregnanolone in the dorsal hippocampus in the elevated plus maze in rats. **(A)** Time spent on open arms. **(B)** Number of entries into open arms. *p < 0.05, vs. VEH and antagonists plus C0.5 and A0.5 in proestrus; ^+^*p* < 0.05, vs. VEH in proestrus; ^#^ *p* < 0.05, vs. VEH and antagonists plus C0.5 and A0.5 plus in dioestrus (Student-Newman-Keuls *post hoc* test). VEH, vehicle; C0.5, 0.5 μg chrysin; P, picrotoxin; B, bicuculline; F, flumazenil; A0.5, 0.5 μg allopregnanolone. The data are expressed as the mean ± standard error of the mean from 7 to 8 rats per group.

The analysis of the time spent on the open arms revealed significant effects of the treatment [*F*_(8_,_121)_ = 8.104, *p* < 0.001] and the oestrous cycle phase [*F*_(1_,_121)_ = 106.512, *p* < 0.001] and a significant treatment × oestrous cycle phase interaction [*F*_(8_,_121)_ = 7.892, *p* < 0.001]. The *post hoc* test showed that the administration of picrotoxin, bicuculline, and flumazenil blocked the decrease in the time spent on the open arms that was produced by 0.5 μg of chrysin and allopregnanolone in proestrus, reaching values that were similar to those in the vehicle-proestrus group. Picrotoxin, bicuculline, and flumazenil prevented the increase in the time spent on the open arms that was produced by 0.5 μg of chrysin and allopregnanolone in dioestrus, reaching values that were similar to the vehicle-dioestrus group (Figure 3A). No significant differences were detected between the effects of the three antagonists on this variable.

The analysis of the number of entries into the open arms showed no effect of treatment [*F*_(8_,_121)_ = 1.450, *p* = 0.183] but a significant effect of the oestrous cycle phase [*F*_(1_,_121)_ = 81.488, *p* < 0.001] and a significant treatment × oestrous cycle phase interaction [*F*_(8_,_121)_ = 14.508, *p* < 0.001]. The *post hoc* test showed that the administration of the three antagonists in proestrus blocked the effect of 0.5 μg of chrysin and allopregnanolone on the number of entries into the open arms, producing values that were similar to those in the vehicle-proestrus group. In dioestrus, the three antagonists blocked the increase in the number of entries into the open arms that was produced by 0.5 μg of chrysin and allopregnanolone, producing values that were similar to those in the vehicle-dioestrus group (Figure 3B). No significant differences were detected between the effects of the different antagonists on this variable.

The results of the closed-arm entries are presented in Supplementary Table 3. The statistical analysis did not reveal significant differences associated with treatments [*F*_(8_,_121)_ = 0.949, *p* = 0.479], the ovarian cycle phase [*F*_(1_,_121)_ = 2.902, *p* = 0.097] and the treatment × oestrous cycle phase interaction [*F*_(8_,_121)_ = 0.883, *p* = 0.433].

### Locomotor Activity Test

The LAT results are presented in Supplementary Table 4. Similar to Experiment 1, none of the variables in this test (i.e., crossings, grooming, and rearing) were significantly affected by the treatments or the ovarian cycle phase.

## Discussion

The present study explored the effects of the dorsal hippocampus in chrysin and allopregnanolone on anxiety-like behaviour associated with low (dioestrus phase) and high (proestrus phase) concentrations of ovarian hormones and the involvement of the GABA_*A*_/benzodiazepine receptor complex in such effects. Thus, this is the first report to show that chrysin, similar to allopregnanolone, regulates anxiety-like behaviour by targeting a brain structure involved in the physiopathology of anxiety (i.e., the hippocampus), which depends on the endocrine state associated with the ovarian cycle. This study contributes to the delineation of the neuroanatomical substrate involved in the anxiolytic-like effects of the flavonoid chrysin.

The EPM is a widely validated unconditioned model of anxiety that is used to study the potential anxiogenic and anxiolytic effects of drugs, their mechanisms of action and their brain targets. In this model, higher anxiety-like behaviour was observed when rats spent less time in the open arms, whereas lower anxiety-like behaviour was observed when rats spent more time in the open arms (Korte and de Boer, 2003). Interestingly, rats are treated with specific doses of anxiolytic drugs that activate the GABAergic system and spend more time in the open arms of the EPM without showing changes in their general locomotor activity (Rodgers et al., 1997). In the present study, 0.25 μg of chrysin and allopregnanolone did not modify the levels of anxiety that were observed in rats in dioestrus and oestrus. Increases in the number of entries into the open arms and time spent on the open arms were brought about by the microinjection of 0.5 μg of chrysin and allopregnanolone in the dorsal hippocampus in dioestrus rats. This behavioural change suggests an anxiolytic-like effect that is similar to that of anxiolytic drugs such as benzodiazepines and their derivatives (Rezvanfard et al., 2009; Ravenelle et al., 2018; Rombolà et al., 2019, 2020).

The anxiolytic effects of chrysin and allopregnanolone in dioestrus rats resemble anxiolysis, which naturally occurs in proestrus. In contrast, microinjections of 0.5 and 1 μg of chrysin and allopregnanolone in proestrus rats blocked the naturally occurring anxiolysis associated with this ovarian cycle phase. These results suggest a switch in the effects of chrysin and allopregnanolone when relatively high doses were microinjected into the dorsal hippocampus; however, they were not associated with sedative or stimulant effects on locomotor activity. One possible explanation for these findings in the EPM is the high plasticity of the GABA_*A*_/benzodiazepine receptor complex in the presence of these ligands. *In vitro* studies have shown that high concentrations of GABAergic agonists may alter the GABA_*A*_ subunit configuration to elicit desensitization and even lead to the inactivation of this receptor subtype (Mathers, 1991; Krall et al., 2015). Both chrysin and allopregnanolone target GABA_*A*_ receptors, and steroid hormone concentrations are high in proestrus (MacKenzie and Maguire, 2014). Thus, the combination of high concentrations of endogenous and exogenous ligands (i.e., chrysin plus endogenous steroids or allopregnanolone plus endogenous steroids) may reduce GABA_*A*_ receptor sensitivity, interfere with naturally occurring proestrus-dependent anxiolysis and block the facilitation of GABA_*A*_-mediated inhibition by exogenous ligands.

The effect of the highest dose of chrysin or allopregnanolone (1 μg) microinjected into the dorsal hippocampus of dioestrus rats supports the hypothesis of the lower sensitivity of the GABA_*A*_ receptor, showing an inverted-U effect and returning anxiety to a level that was observed in vehicle-treated rats in dioestrus. Interestingly, bilateral intrahippocampal microinjections of 0.5 μg of the GABA_*A*_ receptor agonist muscimol (Zhang et al., 2014) and 14 and 20 μg allopregnanolone (Engin and Treit, 2007a), produced anxiety-like behaviour in the EPM in male rats, whereas bilateral microinjections of the lower dose of 0.2 μg/side allopregnanolone in the dorsal intrahippocampus produced anxiolytic-like effects (Mòdol et al., 2011). Altogether, evidence suggests a dose-dependent modulation of anxiolytic-like effects of drugs targeting the GABA_*A*_ receptor. Another alternative explanation is that elevated levels of oestradiol are present during early proestrus, which does not occur during early dioestrus. Such differences in the endocrine milieu may be critical in preventing the synergistic effects of chrysin and allopregnanolone with sex hormones. Ovariectomy causes extremely low levels of sex hormones and elicits a progressive increase in [^3^H] flunitrazepam-specific binding that is linked to GABA_*A*_ receptors, and this increase is reduced by oestradiol treatment (Bossé and Di Paolo, 1996). In cycling female rats, progesterone levels, but not oestradiol levels, positively correlate with bicuculline-induced seizure thresholds (Wilson, 1992), supporting the protective role of progesterone and possibly its metabolites, on the behavioural effects elicited by drugs targeting the GABA_*A*_ receptor. This idea deserves to be evaluated in specific experiments in females treated with chrysin or allopregnanolone and coursing with different oestradiol and/or progesterone levels. The present results suggest that chrysin exerts actions that are similar to the endogenous neurosteroid allopregnanolone, modulating anxiety-like behaviour depending on the dose that is microinjected in the dorsal hippocampus and depending on the endocrine state of females. Higher doses of chrysin, which may induce adverse effects, remain unexplored. Further research should be conducted to evaluate the potential negative or toxic effects.

To avoid false positives or false negatives in anxiety models, possible non-specific motor effects of drugs or surgical manipulations need to be discarded. Closed-arm entries were introduced as a measure of motor activity in the EPM (Rodgers and Johnson, 1995). However, agreement as to the “pure” indicator of locomotor activity index of this variables in the EPM remains ambiguous because it is considered more as an index of protected exploration (Bourin, 2015). Recent studies have included the LAT as a complementary measure of a purer locomotor activity to either discard or identify motor changes associated with experimental manipulations (Rodríguez-Landa et al., 2009; Hernández-López et al., 2017). In the present study, none of the treatments affected the closed-arm entries in the EPM or the motor activity in the LAT (i.e., crossings, rearing, and grooming), thus demonstrating the specificity of anxiolytic-like effects in dioestrus and anxiogenic-like effects in proestrus when chrysin and allopregnanolone were microinjected into the hippocampus. These effects are consistent with those in previous studies that reported no significant effects of anxiolytic drugs, the ovarian cycle, or stereotactic surgery on locomotor activity (Rodríguez-Landa et al., 2009, 2013, 2019; Carro-Juárez et al., 2012; Hernández-López et al., 2017; García-Ríos et al., 2019). On the other hand, intraperitoneal injections of chrysin (2 mg/kg) in dioestrus rats increased grooming behaviour in the LAT (Rodríguez-Landa et al., 2021). This difference with the present study could be associated with the different routes of administration and specific experimental conditions, suggesting that the dorsal hippocampus does not directly participate in the regulation of grooming behaviour elicited by chrysin.

The present findings support the hypothesis that chrysin interacts with the GABAergic system in the dorsal hippocampus, which highly expresses GABA_*A*_ receptors and participates in the regulation of emotional processing, anxiety and neuropharmacological effects of anxiolytic drugs (Farajdokht et al., 2020). Studies have explored the participation of the GABA_*A*_/benzodiazepine receptor complex in the actions of anxiolytic drugs using specific antagonists. Picrotoxin is a non-competitive GABA_*A*_ receptor antagonist that blocks the flux of chloride ions through the receptor channel, decreasing GABAergic function (Olsen, 2006). Through this mechanism, picrotoxin blocks the effects of drugs, such as diazepam, gabapentin and allopregnanolone (Kumar and Goyal, 2007; Kumar et al., 2009; Carro-Juárez et al., 2012). Bicuculline is a competitive antagonist of the gamma-aminobutyric acid recognition site in the GABA_*A*_ receptor. It blocks the activation of this receptor by flavonoids and neurosteroids (Cueto-Ecobedo et al., 2020). Flumazenil is a selective antagonist of the benzodiazepine recognition site of the GABA_*A*_ receptor and interferes with the anxiolytic effects of benzodiazepines and allopregnanolone (Fernández-Guasti and Picazo, 1995; Farajdokht et al., 2020). In the present study, the systemic administration of picrotoxin, bicuculline, and flumazenil completely prevented the anxiolytic-like effects of microinjections of 0.5 μg of chrysin and allopregnanolone in the dorsal hippocampus in dioestrus rats, underscoring the critical role of the GABA_*A*_-benzodiazepine receptor complex in anxiolysis mediated by both drugs. Additionally, all GABAergic antagonists elicited a partial reversal of the anxiogenic-like effect of chrysin and allopregnanolone, which was observed in proestrus rats. Such effects may be explained by the saturation of the GABA_*A*_ receptor by its ligands, leading to changes in its functionality. The present results suggest that other endogenous substances that are associated with the endocrine milieu during proestrus may directly impact the effects of chrysin and allopregnanolone on GABA_*A*_ receptor function. Altogether, these findings indicate that the GABA_*A*_/benzodiazepine receptor complex in the dorsal hippocampus participates in the neuropharmacological effects of chrysin and allopregnanolone on anxiety-like behaviour in female rats. The present findings should prompt further clinical research on chrysin, similar to the neurosteroid allopregnanolone, on its ability to ameliorate neuropsychiatric symptoms in women with low concentrations of steroid hormones (Paul et al., 2020; Pinna, 2020a,b).

The present study had several limitations. The groups in Experiment 2 were combined with groups that received microinjections of a vehicle and 0.5 μg of chrysin and allopregnanolone from Experiment 1. This decision was based on the 3R principles (Russell et al., 2005) to reduce the number of experimental animals. The statistical results validated this decision because between-group comparisons yielded significant differences (*p* < 0.05, *p* < 0.001), providing satisfactory statistical support for the neurobehavioural findings. Supporting this decision, we used antagonist doses previously reported in the literature by our laboratory group to avoid replicated groups. Such doses lack effects *per se* in the EPM and LAT (Rodríguez-Landa et al., 2013, 2019). Furthermore, future studies could be conducted to evaluate the microinjection of GABAergic antagonists in the hippocampus to identify possible local interactions among the GABA_*A*_/benzodiazepine receptor complex, chrysin, or allopregnanolone. Finally, another potential limitation could be the use of unilateral vs. bilateral implants into the dorsal hippocampus, which was decided to avoid a potentially higher lesion in the brain, considering the dimensions of the implanted guide cannula. In support of our design, previous studies have reported anxiolytic- and antidepressant-like effects after the unilateral microinjection of substance P (Carvalho et al., 2008), progesterone (Estrada-Camarena et al., 2002), and allopregnanolone (Rodríguez-Landa et al., 2005, 2009), with no significant differences between the hippocampus side. In the present study, we microinjected chrysin and allopregnanolone into the left (dorsal hippocampus) hemisphere to observe clear anxiolytic-like effects.

## Conclusion

The results of this study confirm that chrysin affects anxiety-like behaviour in females and that it directly affects the dorsal hippocampus, a structure with a key role in affective and emotional behaviour modulation. GABA_*A*_ receptors in the dorsal hippocampus seem to have different degrees of participation in the effects of chrysin on anxiety, which is modulated by the endocrine milieu in females, similar to allopregnanolone. Specifically, hormone conditions in dioestrus favor the anxiolysis elicited by chrysin, in interaction with the different binding sites of GABA_*A*_ receptors. In contrast, the proestrus endocrine milieu leads to anxiogenic effects of this flavonoid, which are partially mediated by the GABA_*A*_/benzodiazepine receptor complex. Altogether, our findings contribute to the knowledge of the neuroanatomical and neuropharmacological bases of the effects of chrysin on anxiety-like behaviour that are associated with hormonal fluctuations during the ovarian cycle phases.

## Data Availability Statement

The raw data supporting the conclusions of this article will be made available by the authors, without undue reservation.

## Ethics Statement

The animal study was reviewed and approved by the Internal Committee for the Care and Use of Laboratory Animals (CICUAL-UV).

## Author Contributions

JFR-L, DS, and FH-L: conceptualisation and methodology. FH-L, LM-M, and ER-D: methodology and formal analysis. JFR-L, BB-M, and DS: data curation. JFR-L, LM-M, and DS: writing manuscript. All authors reviewed, discussed, and approved the final version of the manuscript.

## Conflict of Interest

The authors declare that the research was conducted in the absence of any commercial or financial relationships that could be construed as a potential conflict of interest.

## Publisher’s Note

All claims expressed in this article are solely those of the authors and do not necessarily represent those of their affiliated organizations, or those of the publisher, the editors and the reviewers. Any product that may be evaluated in this article, or claim that may be made by its manufacturer, is not guaranteed or endorsed by the publisher.

## Footnotes

1
https://random.org
