# Supplementary material for: GABAA/Benzodiazepine Receptor Complex in the Dorsal Hippocampus Mediates the Effects of Chrysin on Anxiety-Like Behaviour in Female Rats
Source: Front Behav Neurosci. 2022 Jan 5;15:789557. doi: 10.3389/fnbeh.2021.789557 (PMC8766729; doi:10.3389/fnbeh.2021.789557)
Supplement: Supplementary file 1 [file Data_Sheet_1.PDF]

# *Supplementary Material*

## Supplementary Material 1

### Behavioural tests

The rats were sequentially evaluated in the behavioural test battery, first in the elevated plus maze (5 min), and, subsequently, in the locomotor activity test (5 min). On the test day, the rats were brought to the experimental room at 10:00 AM and left for 1 h to acclimate to the novel surroundings. The behavioural evaluations were performed between 11:00 AM and 2:00 PM. Digital video cameras (Sony DCR-SR42, 40× optical zoom, Carl Zeiss lens) were installed above each apparatus to record the rat activity. Two blinded independent observers measured the behavioural variables using *ex professional* software to record the number and time in seconds of each evaluated variable until > 95% agreement was reached between observers. After each individual test, the apparatus was carefully cleaned with a 15% ethanol solution to remove the scent of the previously evaluated rat to avoid any influence on spontaneous behaviour of the next rat.

#### *Elevated plus maze*

The elevated plus maze consisted of two opposite open and closed arms that were set in a plus configuration. The apparatus was situated in an illuminated room at 40 lux. The dimensions of the open arms were 50 cm length × 10 cm width. The dimensions of the closed arms were 50 cm length × 10 cm width with 40 cm high walls. The entire maze was elevated 50 cm above the floor. To evaluate the effects of the treatments, the rats were individually placed in the centre of the maze, facing an open arm. Three variables were evaluated: (a) number of entries into the open arms, (b) number of entries to closed arms, and (c) time spent on the open arms in seconds. After the elevated plus maze test, the rats underwent the locomotor activity test. Approximately 2 min elapsed between tests. These variables were selected based on previous studies in which these measures were shown to be reliable indicators of experimental anxiety and exploration (Walf, 2007; Rodríguez-Landa et al., 2017, 2019).

#### *Locomotor activity test*

The rats were individually placed in a Plexiglas cage (44 cm length × 33 cm width × 20 cm height). The floor of the cage was delineated into 12 squares (11 cm × 11 cm) to evaluate spontaneous locomotor activity (crossing), grooming, and rearing. At the beginning of the test, the rat was gently placed in one of the corners of the cage. The following variables were measured: (a) number of crossings; a crossing was considered when the rat passed from one square to another with its hind legs, (b) time spent grooming (in seconds), including paw licking, nose/face grooming, head washing, body grooming/scratching, leg licking, and tail/genital grooming, and (c) time spent rearing (in seconds), considered when the rat acquired a vertical posture relative to the cage floor (Guillén-Ruiz et al., 2021).

### References


Guillén-Ruiz, G., Cueto-Escobedo, J., Hernández-López, F., Rivera-Aburto, L.E., Herrera-Huerta. E.V., Rodríguez-Landa JF. (2021). Estrous cycle modulates the anxiogenic effects of caffeine in the elevated plus maze and light/dark box in female rats. *Behav. Brain Res*. 10, 413:113469. doi: 10.1016/j.bbr.2021.113469.

Rodríguez-Landa, J.F., Cueto-Escobedo, J., Puga-Olguín, A., Rivadeneyra-Domínguez, E., Bernal-Morales, B., Herrera-Huerta, E.V., et al. (2017). The phytoestrogen genistein produces similar effects as 17*β*-estradiol on anxiety-like behavior in rats at 12 weeks after ovariectomy. *Biomed. Res. Int*. 2017, 9073816. doi: 10.1155/2017/9073816.

Rodríguez-Landa, J.F., Hernández-López, F., Cueto-Escobedo, J., Herrera-Huerta, E.V., Rivadeneyra-Domínguez, E., Bernal-Morales, B., et al. (2019). Chrysin (5,7-dihydroxyflavone) exerts anxiolytic-like effects through $GABA_A$ receptors in a surgical menopause model in rats. *Biomed. Pharmacother*. 109, 2387-2395. doi: 10.1016/j.biopha.2018.11.111.

Walf, A.A., Frye, C.A. (2007). The use of the elevated plus maze as an assay of anxiety-related behavior in rodents. *Nat. Protoc*. 2, 322-328. doi: 10.1038/nprot.2007.44.

**Supplementary Figure 1**

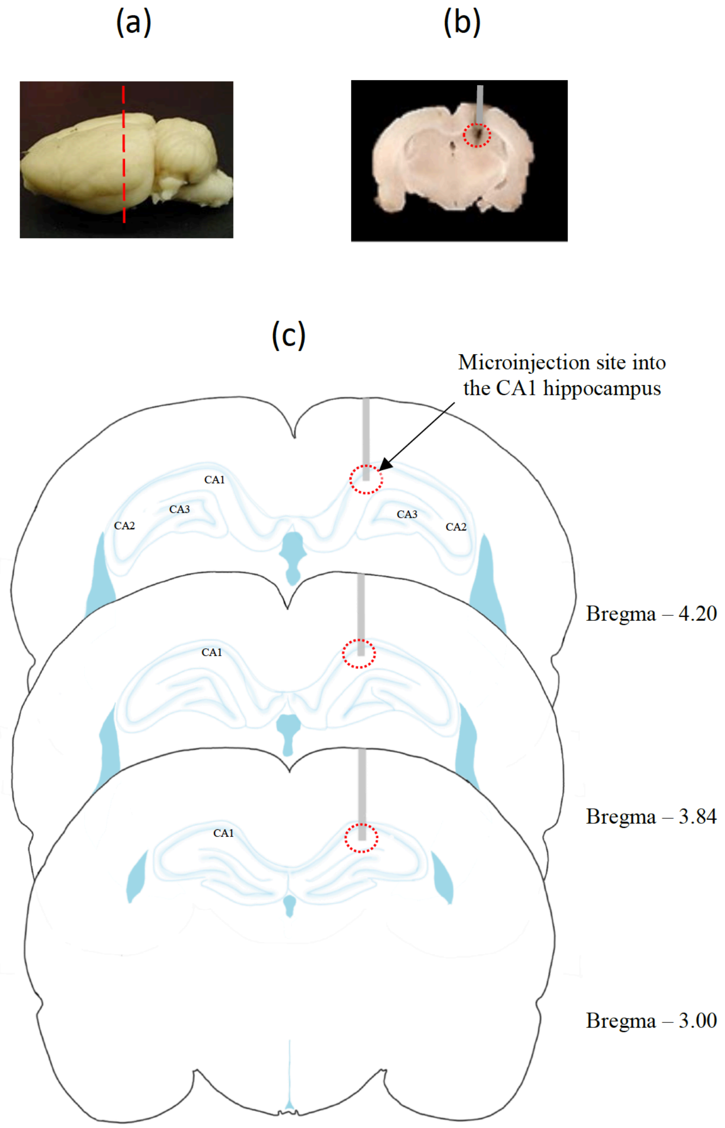


**Supplementary Figure 1**. Representative scheme of the localization of microinjection into the left CA1 area of the dorsal hippocampus. (a) Representative brain rat indicating the level (red dotted line) in which it was sliced. (b) Representative coronal brain section showing the microinjection site (red dotted circle) in CA1 dorsal hippocampus. (c) Coronal brain sections adapted from Paxinos and Watson (2014) rat brain atlas illustrating the dorsal hippocampus in the intended stereotactic coordinates approximately in anterior/posterior, -3.00 to - 4.20 mm from Bregma; medial/lateral, -2.0 mm; dorsal/ventral, -2.0 to -2.5 mm.

Results of closed-arm entries in the EPM are presented in **Supplementary Table 1**. The analysis of this variable did not reveal significant effects of treatment ($F_{6,96} = 0.475$, $p = 0.826$), oestrous cycle phase ($F_{1,96} = 0.350$, $p = 0.556$) and treatment × oestrous cycle phase interaction ($F_{6,96} = 1.927$, $p = 0.084$).

**Supplementary Table 1.** Results of closed-arm entries in the EPM according treatment and ovarian cycle phase in experiment 1.

| Variable/Treatment | Proestrus | Dioestrus |
|---|---|---|
| **Crossing (n)** | | |
| V | 3.2 ± 0.9 | 4.6 ± 0.5 |
| C0.25 | 3.5 ± 0.4 | 3.8 ± 0.5 |
| C0.5 | 4.3 ± 0.6 | 3.4 ± 0.4 |
| C1 | 4.6 ± 0.5 | 3.7 ± 0.4 |
| A0.25 | 3.8 ± 0.3 | 3.6 ± 0.5 |
| A0.5 | 3.7 ± 0.6 | 3.3 ± 0.5 |
| A1 | 3.9 ± 0.3 | 3.4 ± 0.4 |

V, vehicle; C, chrysin; A, allopregnanolone. Treatments of chrysin and allopregnanolone were microinjected in dorsal hippocampus at 0.25, 0.5 and 1 $\mu$g/rat. Data represent the mean ± standard error from 7 to 8 rats per group. Two-way ANOVA.

Results of the evaluated variables in the LAT are presented in **Supplementary Table 2**. The analysis of the number of crossings did not reveal significant effects of treatment ($F_{6,96}$ = 0.302, $p$ = 0.934), oestrus cycle phase ($F_{1,96}$ = 0.080, $p$ = 0.778) and treatment × oestrus cycle phase interaction ($F_{6,96}$ = 1.470, $p$ = 0.196). Similarly, the analysis of the time spent in rearing did not reveal significant effects of treatment ($F_{6,96}$ = 0.239, $p$ = 0.963), oestrus cycle phase ($F_{1,96}$ = 0.303, $p$ = 0.583) and treatment × oestrus cycle phase interaction ($F_{6,96}$ = 1.045, $p$ = 0.401). The analysis of the time spent in grooming did not reveal significant effects of treatment ($F_{6,96}$ = 0.438, $p$ = 0.852), oestrus cycle phase ($F_{1,96}$ = 1.014, $p$ = 0.317) and treatment × oestrus cycle phase interaction ($F_{6,96}$ = 0.210, $p$ = 0.973).

**Supplementary Table 2.** Results of crossing, rearing and grooming in the locomotor activity test according treatment and ovarian cycle phase in experiment 1.

| Variable/Treatment | Proestrus | Dioestrus |
|---|---|---|
| **Crossing (n)** | | |
| V | 54.0 ± 5.4 | 58.5 ± 2.2 |
| C0.25 | 57.1 ± 5.1 | 56.0 ± 3.6 |
| C0.5 | 53.4 ± 5.2 | 50.5 ± 2.6 |
| C1 | 60.2 ± 7.3 | 46.7 ± 3.7 |
| A0.25 | 52.2 ± 4.5 | 61.5 ± 3.7 |
| A0.5 | 57.0 ± 5.2 | 50.1 ± 4.9 |
| A1 | 52.6 ± 4.8 | 58.2 ± 4.7 |
| **Rearing (s)** | | |
| V | 15.1 ± 1.8 | 21.1 ± 1.2 |
| C0.25 | 15.8 ± 2.4 | 19.7 ± 2.5 |
| C0.5 | 18.7 ± 2.6 | 18.6 ± 2.7 |
| C1 | 21.9 ± 2.7 | 18.5 ± 3.2 |
| A0.25 | 20.4 ± 3.5 | 17.3 ± 1.7 |
| A0.5 | 18.2 ± 2.6 | 18.1 ± 2.2 |
| A1 | 18.1 ± 1.8 | 19.7 ± 1.5 |
| **Grooming (s)** | | |
| V | 26.2 ± 2.0 | 28.8 ± 1.9 |
| C0.25 | 27.6 ± 2.2 | 26.6 ± 1.7 |
| C0.5 | 24.8 ± 1.8 | 27.6 ± 2.7 |
| C1 | 29.4 ± 2.4 | 30.2 ± 2.6 |
| A0.25 | 26.2 ± 2.1 | 29.6 ± 2.7 |
| A0.5 | 26.0 ± 1.3 | 27.3 ± 1.6 |
| A1 | 28.3 ± 2.8 | 26.0 ± 1.5 |

V, vehicle; C, chrysin; A, allopregnanolone. Treatments of chrysin and allopregnanolone were microinjected in dorsal hippocampus at 0.25, 0.5 and 1 $\mu$g/rat. Data represent the mean ± standard error from 7 to 8 rats per group. Two-way ANOVA.

Results of closed-arm entries in the EPM are presented in **Supplementary Table 3**. The analysis of this variable did not reveal significant effects of treatment ($F_{8,121} = 0.949$, $p = 0.479$), oestrus cycle phase ($F_{1,121} = 2.902$, $p = 0.097$) and treatment × oestrus cycle phase interaction ($F_{8,121} = 0.883$, $p = 0.433$).

**Supplementary Table 3.** Results of crossing, rearing and grooming in the locomotor activity test according treatment and ovarian cycle phase in experiment 2.

| Variable/Treatment | Proestrus | Dioestrus |
|---|---|---|
| **Crossing (n)** | | |
| V | 3.2 ± 0.9 | 4.6 ± 0.5 |
| C0.5 | 4.3 ± 0.6 | 3.4 ± 0.4 |
| PC0.5 | 3.6 ± 0.5 | 3.2 ± 0.6 |
| BC0.5 | 2.7 ± 0.6 | 4.2 ± 0.9 |
| FC0.5 | 3.3 ± 0.7 | 3.4 ± 0.5 |
| A0.5 | 3.8 ± 0.4 | 3.2 ± 0.5 |
| PA0.5 | 3.5 ± 0.4 | 3.7 ± 0.6 |
| BA0.5 | 3.1 ± 0.7 | 3.8 ± 0.4 |
| FA0.5 | 3.3 ± 0.5 | 4.0 ± 0.8 |

V, vehicle; C, chrysin; A, allopregnanolone; P, picrotoxin; B, bicuculline; F, flumazenil. Treatments of chrysin and allopregnanolone were microinjected in the dorsal hippocampus at 0.5 $\mu$g/rat. Treatments con picrotoxin, bicuculline and flumazenil were i.p. injected at 1, 1 and 5 mg/kg, respectively, 30 min before the microinjection. Data represent the mean ± standard error from 7 to 8 rats per group. Two-way ANOVA.

**Table 4**. The analysis of the number of crossings did not reveal significant effects of treatment ($F_{8,121}$ = 1.202, $p$ = 0.304), oestrus cycle phase ($F_{1,121}$ = 0.0009, $p$ = 0.975) and treatment × oestrus cycle phase interaction ($F_{8,121}$ = 1.062, $p$ = 0.394). Similarly, the analysis of the time spent in rearing did not reveal significant effects of treatment ($F_{8,121}$ = 0.432, $p$ = 0.900), oestrus cycle phase ($F_{1,121}$ = 0.043, $p$ = 0.835) and treatment × oestrus cycle phase interaction ($F_{8,121}$ = 1.510, $p$ = 0.160). In addition, the analysis of the time spent in grooming did not reveal significant effects of treatment ($F_{8,121}$ = 0.798, $p$ = 0.606), oestrus cycle phase ($F_{1,121}$ = 2.241, $p$ = 0.137) and treatment × oestrus cycle phase interaction ($F_{8,121}$ = 0.437, $p$ = 0.897).

**Supplementary Table 4.** Results of crossing, rearing and grooming in the locomotor activity test according treatment and ovarian cycle phase in experiment 2.

| Variable/Treatment | Proestrus | Dioestrus |
|---|---|---|
| **Crossing (n)** | | |
| V | 54.0 ± 5.4 | 58.5 ± 2.2 |
| C0.5 | 53.4 ± 5.2 | 50.5 ± 2.6 |
| PC0.5 | 47.5 ± 2.4 | 48.3 ± 2.3 |
| BC0.5 | 45.8 ± 4.1 | 54.3 ± 2.1 |
| FC0.5 | 49.6 ± 3.7 | 49.6 ± 3.1 |
| A0.5 | 57.0 ± 5.2 | 50.1 ± 4.9 |
| PA0.5 | 56.3 ± 3.3 | 50.3 ± 3.1 |
| BA0.5 | 51.7 ± 3.8 | 47.3 ± 2.1 |
| FA0.5 | 48.5 ± 3.1 | 46.8 ± 2.3 |
| **Rearing (s)** | | |
| V | 15.1 ± 1.8 | 21.1 ± 1.2 |
| C0.5 | 18.7 ± 2.6 | 18.6 ± 2.7 |
| PC0.5 | 18.5 ± 0.8 | 18.0 ± 1.1 |
| BC0.5 | 16.6 ± 0.9 | 18.5 ± 1.2 |
| FC0.5 | 18.7 ± 1.1 | 16.7 ± 1.4 |
| A0.5 | 18.2 ± 2.6 | 18.1 ± 2.2 |
| PA0.5 | 21.7 ± 1.9 | 17.9 ± 2.1 |
| BA0.5 | 19.4 ± 1.1 | 19.5 ± 1.9 |
| FA0.5 | 20.2 ± 1.2 | 17.8 ± 1.0 |
| **Grooming (s)** | | |
| V | 26.2 ± 2.0 | 28.8 ± 1.9 |
| C0.5 | 24.8 ± 1.8 | 27.6 ± 2.7 |
| PC0.5 | 22.9 ± 2.8 | 25.4 ± 2.1 |
| BC0.5 | 26.7 ± 2.1 | 26.9 ± 2.4 |
| FC0.5 | 24.2 ± 1.6 | 24.6 ± 1.9 |
| A0.5 | 26.0 ± 1.3 | 27.3 ± 1.6 |
| PA0.5 | 25.9 ± 2.4 | 24.1 ± 1.7 |
| BA0.5 | 25.5 ± 2.7 | 29.4 ± 1.4 |
| FA0.5 | 26.9 ± 2.4 | 276 ± 2.2 |

V, vehicle; C, chrysin; A, allopregnanolone; P, picrotoxin; B, bicuculline; F, flumazenil. Treatments of chrysin and allopregnanolone were microinjected in the dorsal hippocampus at 0.5 $\mu$g/rat. Treatments con picrotoxin, bicuculline and flumazenil were i.p. injected at 1, 1 and 5 mg/kg, respectively, 30 min before the microinjection. Data represent the mean ± standard error from 7 to 8 rats per group. Two-way ANOVA.
